# Diet and Nutrtion: Sunny Side of Cancer Prevention

**DOI:** 10.1289/ehp.115-a402b

**Published:** 2007-08

**Authors:** M. Nathaniel Mead

Many studies have linked higher latitudes with greater breast cancer risk, and seasonal variations are now thought to influence cancer incidence and mortality. The pivotal mediators in these relationships are believed to be sunshine and vitamin D. In clinical studies, poor vitamin D status is associated with a substantial increase in breast cancer incidence and mortality, though until recently, there has been only limited evidence in humans that vitamin D–related factors may reduce breast cancer risk. New studies are now clarifying the role of this nutrient.

For most people, sunshine is the main source of vitamin D and specifically of 25(OH)D. Vitamin D is unusual among nutrients because very few foods naturally contain it, and supplementation is intended primarily to compensate for a deficiency of sunshine. Most human photosynthesis occurs in the summer, because the angle of sunlight largely determines the rate of vitamin D synthesis. Among the better-defined mechanisms of vitamin D’s anticancer action are its ability to induce cell differentiation and apoptosis of cancer cells while also inhibiting cell proliferation, angiogenesis, and metastasis.

In the March 2007 issue of *Cancer Epidemiology Biomarkers & Prevention*, authors from Mount Sinai Hospital in Toronto described a case–control study involving 972 women with newly diagnosed invasive breast cancer and 1,135 healthy controls. All participants were interviewed to assess vitamin D–related exposures, such as outdoor activities, sun exposure habits (e.g., use of sun-screen), and dietary contributions (cod liver oil and milk consumption). More frequent sun exposure during adolescence was associated with a 35% reduction in breast cancer risk later in life. Lower risk was also linked to cod liver oil intake and drinking at least 10 glasses of milk per week. Milder protection was seen for people aged 20 to 29, but not for people over age 45.

“Our study suggests that vitamin D–related exposures during adolescence and early adulthood, when the breasts are developing, may be more important than later exposure,” says lead author Julia Knight, a senior investigator at Toronto’s Prosserman Centre for Health Research. “A study in postmenopausal women asking about recent diet or sun would miss an effect of earlier exposure.”

Knight is also interested in knowing what effect sunlight supplementation may have in women following treatment for breast cancer. An epidemiologic study of different regions of Norway, each with a different annual UV exposure, found that the prognosis was 15–25% better for women diagnosed (and thus treated) in the summer versus the winter. This research was published in the May 2007 issue of *Breast Cancer Research and Treatment*.

A second study out of Laval University in Quebec sought to assess seasonal variations of breast density, a biomarker of breast cancer risk. The goal was to compare such variation, if any, with blood levels of 25(OH)D. It was assumed that a delay or lag time might be likely between a change in 25(OH)D levels and the resulting change in breast density. The researchers recruited 741 premenopausal women at screening mammography and found a strong inverse correlation between mammographic breast density and circulating vitamin D levels. When a four-month lag time was assumed, seasonal variations of breast density and circulating vitamin D appeared to be highly synchronized, so much so that “variations of breast density appeared to be a mirror image of those of 25(OH)D,” the researchers wrote in the May 2007 issue of *Cancer Epidemiology Biomarkers & Prevention*.

“Taking seasonal variations and lag time into account appears to be crucial when studying the relation of circulating vitamin D to breast density,” says lead author Jacques Brisson, a professor in the Department of Social and Preventive Medicine at Laval University. “These time-related variables may also be crucial when studying the effect of vitamin D supplementation, because the latter is likely to vary with time, typically being more pronounced in fall and winter, for example.”

Brisson and Knight agree that the next step is to carry out randomized trials of the effect of vitamin D supplementation, as only this type of design would establish causality between high vitamin D intake and reduction in breast cancer risk. A randomized controlled study published in the June 2007 issue of the *American Journal of Clinical Nutrition* found that women who regularly took vitamin D_3_ and calcium had a 60% reduction in all-cancer incidence compared with a group taking placebos and a 77% reduction when the analysis was confined to cancers diagnosed after the first 12 months. Observational studies such as those spearheaded by Brisson and Knight have suggested a less dramatic reduction in cancer risk (50% or more).

Public health officials agree that limiting sun exposure will reduce the rate of skin cancers, but some scientists worry that such advice may lead to undesirable tradeoffs on a massive scale. “There are at most one or two extra skin cancer deaths [per hundred thousand people] when you compare the northern to southern United States . . . versus about thirty or forty fewer deaths for the other major cancers,” says Reinhold Vieth, an associate professor of nutrition at the University of Toronto. “So if you tally up the number of deaths likely to be attributable to UV light or vitamin D, it does not look like the most sensible policy to tell people to keep out of the sun just to prevent skin cancer.” Vieth’s advice is for white people to expose more skin for a shorter time, for example by lying in a bathing suit for 10 minutes on each side, twice per week, and over 4 to 8 weeks of summer. Darker-skinned people should stay in the sun longer or take vitamin D supplements.

